# Chronic High-Altitude Hypoxia Alters Iron and Nitric Oxide Homeostasis in Fetal and Maternal Sheep Blood and Aorta

**DOI:** 10.3390/antiox11091821

**Published:** 2022-09-15

**Authors:** Taiming Liu, Meijuan Zhang, Avoumia Mourkus, Hobe Schroeder, Lubo Zhang, Gordon G. Power, Arlin B. Blood

**Affiliations:** 1 Department of Pediatrics, Loma Linda University School of Medicine, Loma Linda, CA 92354, USA; 2 Lawrence D. Longo Center for Perinatal Biology, Loma Linda University School of Medicine, Loma Linda, CA 92354, USA

**Keywords:** chronic hypoxia, pregnancy, oxidative stress, placenta, chemiluminescence nitric oxide (NO) measurement, electron paramagnetic resonance (EPR)

## Abstract

The mammalian fetus thrives at oxygen tensions much lower than those of adults. Gestation at high altitude superimposes hypoxic stresses on the fetus resulting in increased erythropoiesis. We hypothesized that chronic hypoxia at high altitude alters the homeostasis of iron and bioactive nitric oxide metabolites (NOx) in gestation. To test for this, electron paramagnetic resonance was used to provide unique measurements of iron, metalloproteins, and free radicals in the blood and aorta of fetal and maternal sheep from either high or low altitudes (3801 or 300 m). Using ozone-based chemiluminescence with selectivity for various NOx species, we determined the NOx levels in these samples immediately after collection. These experiments demonstrated a systemic redistribution of iron in high altitude fetuses as manifested by a decrease in both chelatable and total iron in the aorta and an increase in non-transferrin bound iron and total iron in plasma. Likewise, high altitude altered the redox status diversely in fetal blood and aorta. This study also found significant increases in blood and aortic tissue NOx in fetuses and mothers at high altitude. In addition, gradients in NOx concentrations observed between fetus and mother, umbilical artery and vein, and plasma and RBCs demonstrated complex dynamic homeostasis of NOx among these circulatory compartments, such as placental generation and efflux as well as fetal consumption of iron-nitrosyls in RBCs, probably HbNO. In conclusion, these results may suggest the utilization of iron from non-hematopoietic tissues iron for erythropoiesis in the fetus and increased NO bioavailability in response to chronic hypoxic stress at high altitude during gestation.

## 1. Introduction

Hypoxia, a condition of insufficient oxygen availability, is one of the most common and severe stresses to an organism’s maintenance of homeostasis. Partial pressure of oxygen (PO_2_) decreases with increasing elevation in altitude, such that high altitude can result in hypoxia that leads to various complex physiological adaptations such as redistribution of cardiac output and increased erythropoiesis to improve oxygen delivery [1]. The fetus lives in an environment where the oxygen availability is much lower than that of the adult [2]. Even at sea level, arterial PO_2_ is ~25 mmHg in a fetus versus ~100 mmHg in an adult. While fetal adaptations of increased cardiac output and hemoglobin O_2_ affinity enable tissue O_2_ delivery to be similar to that of the adult, normal fetal tissue PO_2_ at sea level are comparable to those of an adult at the top of Mount Everest. However, it is well known that the perinatal period presents an extreme challenge to both the mother and offspring, with the transition from fetus to newborn testing the physiological limits of both the newborn and mother [1,2,3,4]. The superimposed hypoxia of high altitudes during pregnancy puts stress on the fetus which can lead to growth restriction and various lifelong sequelae such as hypertension, obesity, diabetes, and cognitive and behavioral disorders [1]. In addition, pregnancy at high altitude also places mothers at higher risk of illness, such as a 2- to 4-fold increased risk of preeclampsia at high altitude compared to low altitude [3]. Nevertheless, although there are >81 million people living at high altitude (>2500 m) [4], our understanding of the mechanisms underlying the impacts of high altitude on gestation is still very limited [1]. 

As a vital component of hemoglobin and many enzymes, iron is essential for the oxygenation and normal function of cells and its concentrations are under tight regulation. For example, the intracellular chelatable iron pool (CIP), defined methodologically as iron that is accessible to chelators, is maintained at low levels by many mechanisms such as ferroportin, the only known cellular iron exporter. Ferroportin, in turn, is suppressed by the hormonal iron regulator hepcidin. The plasma analogue of the CIP, non-transferrin bound iron (NTBI), is oxidized from Fe^2+^ to Fe^3+^ by ceruloplasmin, a multicopper oxidase, and can then be captured by circulating transferrin for cell uptake. Iron in both the CIP and NTBI can catalyze the production of reactive oxygen (ROS), nitrogen (RNS), and sulfur (RSS) species from oxygen, nitric oxide, and hydrogen sulfide, respectively [5,6]. As such, the CIP and NTBI are maintained at the lowest sufficient levels under physiological conditions [6,7]. Given that the body’s largest pool of iron, hemoglobin, is essential for oxygen delivery, it is perhaps not surprising that hypoxia affects iron homeostasis at many levels. For example, ferroportin, ceruloplasmin, and hepcidin are all under the regulation of hypoxia inducible factor [8]. During pregnancy, iron regulatory mechanisms must adapt to supply the growing fetus with large quantities of iron, putting the mother at risk of iron deficiency if dietary supplementation is not provided [9]. How chronic hypoxic stress affects iron homeostasis in the fetus and mother, which have a simultaneously competitive and cooperative relationship with regards to resource allocation [10], remains largely unclear.

Like iron, nitric oxide (NO) is also of significant developmental and biological importance [11]. NO is a reactive free radical that is rapidly metabolized (biological t_1/2_ < 2 s) into various NO species (NOx), such as nitrite, nitrate, S-nitrosothiols (SNOs), and iron-nitrosyls (FeNO) including hemoglobin-NO (HbNO) and dinitrosyl iron complexes (DNICs) [12,13]. These NOx are of differing abundances, bioactivities, and stabilities. For instance, nitrate accounts for >95% of total NOx in plasma but is a poor indicator of endothelial NO synthase (NOS) activity, whereas plasma nitrite reflects the endothelial function [14]. SNOs and DNICs, which are as vasoactive as NO per se, are relatively unstable compared to nitrate and nitrite and thus may vanish during sample collection and processing [15,16,17]. For example, while DNICs have been proposed as the most abundant intracellular NOx [18], they have been largely overlooked in previous studies due to challenges in detecting them before they degrade to nitrite or nitrate [15,16,17]. Of relevance to the current studies, DNICs are also involved in sequestration and export of both intracellular iron and nitric oxide [18,19]. 

Oxygen plays a complex role in regulating both the production and metabolism of NO [20]. For example, O_2_ is a necessary substrate for the production of NO by NOS, and thus NO production can be limited under hypoxic conditions in accordance with the Km of NOS enzymes for O_2_ (eNOS = 23 µM, iNOS = 135 µM, nNOS = 350 µM). In addition, NO reacts avidly with O_2_ that is bound to heme proteins such as oxyhemoglobin to produce nitrate, or with free O_2_ to form nitrite, which tends to lower NO concentrations under normoxic or hyperoxic conditions. Conversely, under hypoxic conditions NOS-independent pathways for NO production become important, such as the conversion of nitrite to NO by reaction with heme-containing proteins [21]. How high-altitude hypoxia affects NO homeostasis, especially in the mother and fetus, remains largely unknown. Although enhanced production of NO, debatably via upregulation of eNOS, has been proposed as a universal response to hypoxic stress [22,23,24,25], most previous studies of the effects of high-altitude hypoxia on NO homeostasis have been limited by the need to freeze and process samples in a way that fails to preserve the most bioactive NOx species and thus most have reported nitrate concentrations, which is a poor indicator of overall NO bioactivity [14]. Nevertheless, high-altitude has been associated with a decrease rather than an increase in plasma nitrate concentrations in pregnant women [26]. In addition, we recently demonstrated an efflux of FeNOs from the placenta to fetal plasma in sheep, further raising the question of what role NOx metabolites play in fetal physiology, and whether NO homeostasis adapts to high-altitude in gestation [27]. Therefore, further investigation of the effects of high-altitude hypoxia on NOx in gestation with the focus on the bioactive NOx is needed.

The current study hypothesizes that chronic hypoxia at high-altitude alters fetal and maternal iron and NO homeostasis. To test this, we used electron paramagnetic resonance (EPR) to measure iron, metalloproteins, and free radicals in blood and homogenized aortas of fetal and maternal sheep maintained at either high or low altitude (3801 and 300 m). In addition, using chemiluminescent NOx measurement methodology capable of distinguishing between various NOx species [28], this study determined the concentrations of bioactive NOx in these samples immediately after collection.

## 2. Materials and Methods

### 2.1. Experimental Animals

Animal protocols (IACUC#8200001 approved on 9 February 2020 for the period of 9 February 2020 to 30 January 2023) were preapproved by the Institutional Animal Care and Use Committee of Loma Linda University and were in accordance with guidelines of the American Physiologic Society and the National Institutes of Health. The current study used blood and thoracic aorta collected from two-year old pregnant ewes and their fetuses at about 140 days gestation (term = ~150 days). High altitude animals maintained at 3801 m for the latter ~90 days before study [29] were compared to low altitude (300 m) controls.

For each experiment, five to six sheep were used in each experimental group. For the low-altitude group (normoxic), sheep were maintained at the supplier’s ranch (Nebeker Ranch Inc. Lancaster, CA, USA) on alfalfa pellets ad libitum. For the high-altitude group (long-term hypoxic), ewes at ~50 days gestation were transported from low altitude to the Barcroft Laboratory on White Mountain (Bishop, CA, USA; 3801 m elevation, barometric pressure −480 mmHg), where they were kept until 135 days gestation (near term) in an outdoor sheltered pen and were fed with alfalfa pellets ad libitum. Sheep from both groups were kept in natural day-night conditions. In previous studies [1,29], mean maternal arterial blood gas values from 12 adult sheep, of the same breed and age as those used in the present study, while at high altitude, were PO_2_ = 60 ± 5 mmHg, PCO_2_ = 30.0 ± 2.5 mmHg, and pH = 7.36 ± 0.06. In contrast, normoxic low-altitude sheep had arterial PO_2_ of 100 ± 5 mmHg, PCO_2_ = 35.2 ± 0.9 mmHg, and pH = 7.44 ± 0.1. With high altitude exposure, fetal arterial PO_2_ fell from 25 ± 1 to 19 ± 1 mmHg [1,29]. 

At ~135 days gestation, ewes from both groups were transported (a 6 to 7 h trip for the high-altitude animals) to the laboratory at Loma Linda University. As previously described [1], in order to maintain systemic hypoxia in the high-altitude animals while they awaited study, soon after the arrival to the laboratory, the ewes were surgically instrumented with a tracheal catheter for administration of humidified N_2_ gas and a femoral arterial catheter for sampling arterial blood gases. The flow of N_2_ gas into the trachea was adjusted to lower inspired PO_2_ to a level that maintained the arterial PO_2_ at ~60 Torr until study (≤4 days). At the time of sample collection, the N_2_ flow was discontinued and the ewes breathed room air for ~4 min before anesthesia. In both groups, the ewes were anesthetized with thiopental sodium (10 mg·kg^−1^, i.v.), and anesthesia was maintained with inhalation of 1.5% isoflurane in 100% oxygen throughout sample collection. The fetuses were delivered via C-section. After collection of blood samples and euthanization of both the ewe and fetus by thoracotomy and removal of the hearts, the thoracic aortas were collected. 

### 2.2. Sample Collection and Preparation

Blood samples were collected at around 8 am after overnight fasting with heparinized Monoject^TM^ Rigid Pack syringes (Dublin, OH, USA). In both high- and low-altitude groups, the adult venous whole blood (AVWB) was collected from the jugular vein of the ewe right before anesthesia (high-altitude ewes breathed room air for ~3 min). Fetal whole blood was collected from the umbilical cord artery (UAWB; deoxygenated blood from fetus) and vein (UVWB; oxygenated blood from placenta) after Cesarean section and before cord clamping.

Plasma was separated from red blood cells immediately after blood collection by centrifugation in Eppendorf tubes (Hamburg, Germany) at 6740× *g* for 30 s. The red blood cells were washed three times with cold saline and then reconstituted with saline to the original volume of the whole blood sample, with the final product named RBC. One aliquot of each of the whole blood (WB), plasma (P), and RBC samples was snap frozen in an EPR tube. Whole blood and plasma were also analyzed immediately for NOx concentrations via chemiluminescence. To guarantee that these samples could be analyzed within 4 min of blood collection, two or three investigators (not including the technicians caring for the sheep) were assigned to work together on sample collection, processing, and detection.

Isolated thoracic aorta was cleaned of blood with gauze and ice-cold HEPES buffer. Within 2 h of collection, it was homogenized using a rotor-stator homogenizer (TissueRuptor, Qiagen Inc.; Hilden, Germany) in ice-cold HEPES buffer (1 g in 5 mL) and then centrifuged at 2200× *g* for 15 min. This supernatant was divided into three aliquots. One aliquot was snap frozen in an Eppendorf tube for analysis of NOx. Two aliquots received a final concentration of 1 mM deferoxamine (DFO) or a similar volume of saline, respectively, incubated at 37 °C for 4 h, and snap frozen in EPR tubes. Samples were then stored at −80 °C until assayed.

### 2.3. Analytical Methods

**Figure 1:**
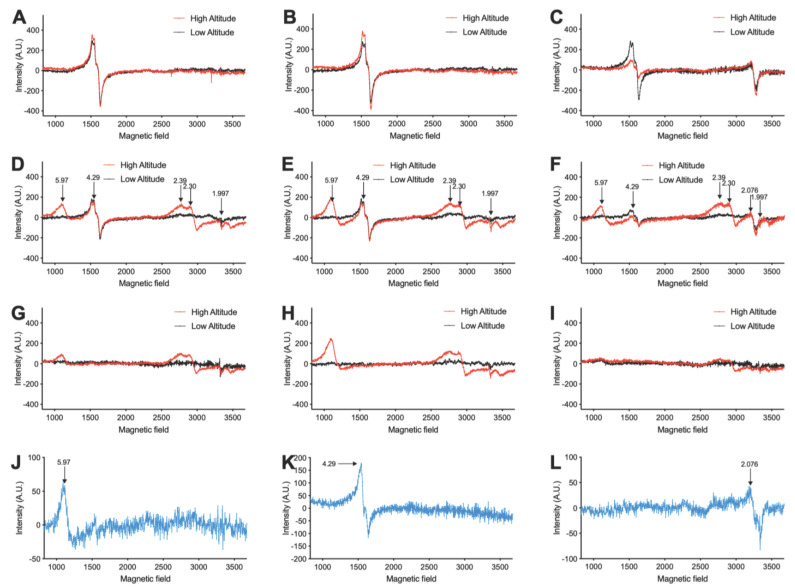
Representative EPR spectrum of blood from high- and low-altitude pregnant sheep. (**A**–**C**) Plasma. (**D**–**F**) Whole blood. (**G**–**I**) Washed RBCs. (**A**,**D**,**G**) Blood from umbilical vein. (**B**,**E**,**H**) Blood from the umbilical artery. (**C**,**F**,**I**) Blood from maternal jugular vein. (**J**–**L**) Standards of metHb (60 μM), transferrin (14.5 μM), and ceruloplasmin (2.3 μM), respectively. Arrows indicate g factors: 5.97 for metHb, 4.29 for transferrin, 2.39 and 2.30 for ferric heme proteins, 2.076 for ceruloplasmin, and 1.997 for free radicals.

EPR signals were recorded at 150 K using a Bruker X-Band EMX Plus EPR spectrometer with a cavity of high sensitivity as previously described [15]. The EPR was set to a microwave power of 20 mW, microwave frequency of 9.31 GHz, attenuator of 10 dB, modulation amplitude of 5 G, modulation frequency of 100 kHz, time constant of 10.24 msec, conversion time of 40.96 msec, harmonic of 1, and scan number of 2. Standard curves for metHb, transferrin (holo), and ceruloplasmin were prepared by spiking different concentrations of standards into HEPES buffer. Interpretation of the EPR spectra was based on comparison of the position and structure of the resonance lines with those reported previously [30,31,32,33,34,35] and with the standards shown in Figure 1J–L.

The CIP was detected with use of the chelator deferoxamine (DFO), which combines with chelatable iron to give an EPR signal of DFO-iron at g = 4.33. Because the CIP signal overlaps with that of ferritin, which appears in the absence of DFO [36], the latter was subtracted before calculation of the CIP concentration. The concentration of the CIP was calculated by comparison with the signal amplitude of known concentrations of DFO-iron standards as previously described [37].

NO metabolite (NOx) levels were measured by four different assays with an ozone-based chemiluminescence NO analyzer (280i, Sievers, Boulder, CO, USA) as previously described [28]. In each assay, the sample is injected into a purge vessel containing one of four reagents known to selectively convert various combinations of NOx species into free NO gas to be carried into the NO analyzer by sparging with argon. The four assay reagents used were: (1) triiodide (I_3_; measures NO, nitrite, SNO, DNIC, and HbNO), (2) ferricyanide + ascorbic acid (FeCN/HAc; measures NO, nitrite, DNIC, and HbNO), (3) ferricyanide + PBS (FeCN/PBS; measures NO, DNIC, and HbNO), and (4) ascorbic acid + acetic acid (VitC/HAc; measures NO, nitrite, and HbNO). All reagents were made freshly on the day of experiment. After each injection, the purge vessel was rinsed with saline and the reagents were replaced. Notably, none of these assays detect nitrate. To minimize changes from metabolism during sample handling, all whole blood and plasma samples were measured within 2 to 4 min of blood collection, with each assay completed in 5 to 6 sheep.

Total iron in plasma and aortic homogenates were measured using a colorimetric ferrozine assay as previously described [38]. Briefly, 100 μL of sample was mixed with 100 μL of 10 mM HCl, and 100 μL of the iron-releasing reagent (a freshly mixed solution of equal volumes of 1.4 M HCl and 4.5% (*w*/*v*) KMnO_4_ in H_2_O) for 2 h at 60 °C. After the mixtures were cooled to room temperature, 30 μL of the iron-detection reagent (6.5 mM ferrozine, 6.5 mM neocuproine, 2.5 M ammonium acetate, and 1 M ascorbic acid dissolved in water) was added. After 30 min, 250 μL of the solution was transferred into a 96-well plate and the absorbance was measured at 550 nm on a microplate reader. Iron concentrations were estimated by comparing the absorbance of the sample to that of the known standards of FeCl_3_ (mixture of 100 μL of FeCl_3_ standards (0–300 μM) in 10 mM HCl, 100 μL HEPES buffer, 100 μL releasing reagent, and 30 μL detection reagent).

Non-transferrin-bound iron (NTBI) in plasma was measured using a commercially available kit (BioVision; Milpitas, CA, USA). ELISA kits were used to measure plasma hepcidin (Biosource; San Diego, CA, USA), and ferroportin (Biosource; San Diego, CA, USA) and nitrotyrosine (Millipore; Temecula, CA, USA) in aortic homogenates. The antioxidant capacity was also measured using a kit that measures the antioxidant activity as the equivalent of Trolox^TM^ in the suppression of the radical formation from the reaction of metmyoglobin and H_2_O_2_ (Sigma-Aldrich; St. Louis, MO, USA).

### 2.4. Statistics

Average values are given as mean ± SEM in the text and figures. One-way ANOVA, two-way ANOVA, linear regression, and *t* tests were used as indicated in the figure legends following normality tests. Repeated measures were used when applicable as specified in the figure legends. Statistical analyses were carried out with Prism, v8.4.0 (Graphpad Software, La Jolla, CA, USA) with significance accepted at *p* < 0.05.

## 3. Results

### 3.1. High Altitude-Induced Alterations of Metalloproteins, Free Radicals, and Iron Levels in Fetal and Maternal Sheep Blood

**Figure 2:**
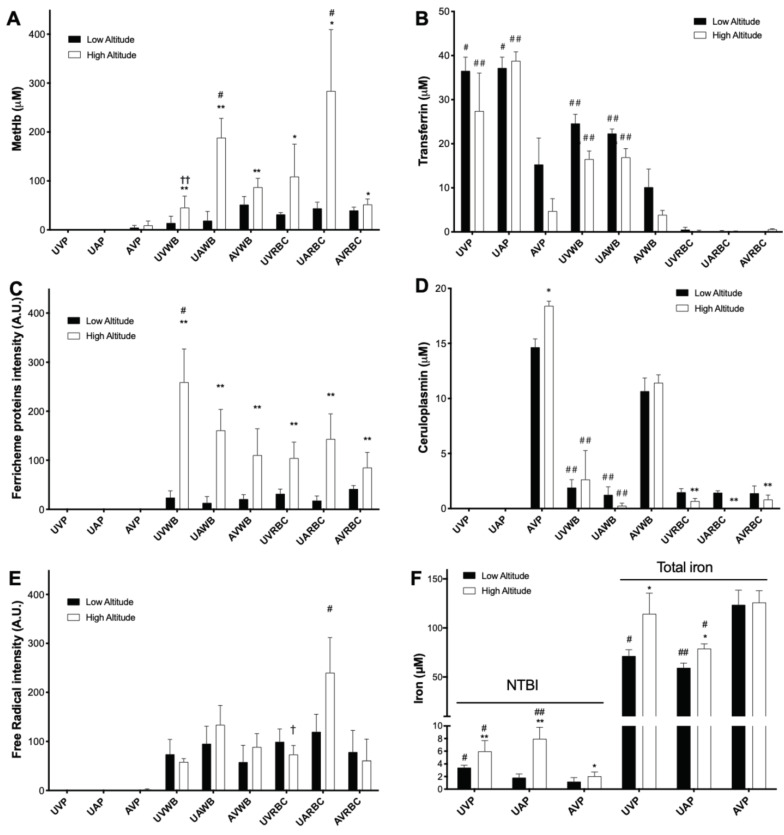
Metalloproteins, free radicals, and iron in blood from high and low altitude pregnant sheep. (**A**–**D**) Metalloproteins quantified from EPR spectra. (**A**) MetHb. (**B**) Transferrin. (**C**) Ferric heme proteins. (**D**) Ceruloplasmin. (**E**) Free radicals quantified from EPR spectra. (**F**) Plasma NTBI and total iron quantified with colorimetric methods. Because of the lack of standards, the intensity of the EPR signal of ferric heme proteins and free radicals are shown. UVP: umbilical vein plasma; UAP: umbilical artery plasma; AVP: adult (maternal) vein plasma; WB: whole blood; RBC: washed red blood cells. Two-way ANOVA with Tukey’s post-hoc tests for P, WB, or RBCs. * = *p* < 0.05 high vs. low altitude; † = *p* < 0.05 umbilical artery vs. vein; # = *p* < 0.05 fetus vs. mother. One symbol = *p* < 0.05, two symbols = *p* < 0.01.

Figure 1A–I shows the representative EPR spectra of blood samples from fetal and maternal sheep from low and high altitude. Paramagnetic centers with g factors of 5.97, 4.29, 2.39–2.30, 2.076, and 1.997 were assigned to metHb, transferrin, ferric heme proteins, ceruloplasmin, and free radicals, respectively [30,31,32,33,34,35]. Their quantified intensities are shown in Figure 2A–E. Levels of NTBI and total iron in plasma are shown in Figure 2F.

Compared with low altitude, high altitude significantly increased metHb and ferric heme proteins, oxidation products of hemes, in both fetuses and mothers, indicating increases of oxidative stresses caused by high altitude hypoxia (Figure 2A,C). Consistent with these results, the plasma NTBI, a marker of oxidative stress, was increased by high altitude in both the fetus and mother (Figure 1F). Notably, the levels of metHb, NTBI, and free radical in umbilical arterial blood (venous blood from fetus) are higher than that in maternal venous blood (Figure 2A,E,F). Together, these results suggest that high altitude hypoxia results in oxidative stress during gestation, especially in the fetus.

Whole blood levels of transferrin in both fetus and mother were decreased by high altitude (Figure 2B). In addition, while consistent with the previous work showing that fetal sheep plasma is deficient in ceruloplasmin [39], the current study found ceruloplasmin in the fraction of washed fetal RBCs, possibly bound to the RBC membrane [40,41] (Figure 2D). In addition, the RBC ceruloplasmin in both fetus and mother was decreased by high altitude. The altered levels of circulating metalloproteins important for iron metabolism, together with the increased NTBI and total iron in fetal plasma (Figure 2F), demonstrate alterations in circulatory iron homeostasis caused by high altitude hypoxia during gestation.

### 3.2. High Altitude Decreases Iron in Fetal and Maternal Aorta

**Figure 3:**
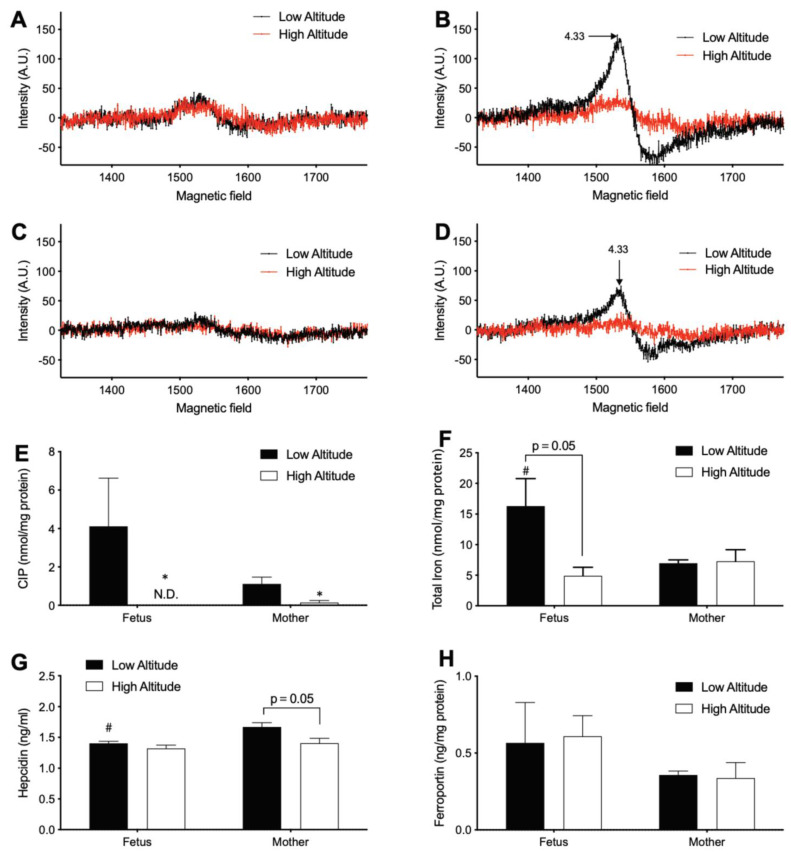
Iron in the aorta of high and low altitude pregnant sheep. (**A**–**D**) Representative EPR spectrum of aorta. (**A**) Fetal aorta. (**B**) Fetal aorta + DFO. (**C**) Mother aorta. (**D**) Mother aorta + DFO. (**E**) CIP in aorta. (**F**) Total metal iron. (**G**) Hepcidin in plasma. (**H**) Ferroportin in aorta. N.D.: not detectable. Two-way ANOVA with Sidak’s post-hoc tests. * = *p* < 0.05 high vs. low altitude; # = *p* < 0.05 fetus vs. mother. *p* value in (**F**) for *t*-test.

Next, the effects of high altitude on iron homeostasis in the fetal and maternal aorta, a representative non-hematopoietic tissue (Figure 3), were measured. At sea level, both the CIP and total iron concentrations in the aorta of the fetus were about twice the levels of the mother (Figure 3E,F), suggesting a higher steady-state level of iron homeostasis in the fetus than the mother. The aortic CIP decreased with high altitude in both fetus and mother significantly to levels that were hardly detectable (Figure 3A–E), suggesting chronic hypoxia resulted in a significant alteration of cellular iron handling. The aortic total iron level in the fetus was decreased (*p* = 0.05) by high altitude, although no significant change was observed in the mother (Figure 3F). These results are suggestive of iron deficiency in the high-altitude fetal aorta. To test whether the iron deficiency results from a decreased supply of iron from the mother and/or increased export of iron, we measured the level of the plasma hepcidin, which in the maternal plasma negatively regulates the iron transport across the placenta to the fetus [42]. This work also measured the level of aortic ferroportin, the only known cellular iron exporter [43]. Contrary to either of these possibilities, high altitude decreased plasma hepcidin levels in only the mother (Figure 3G) and did not alter aortic ferroportin in either mother or fetus (Figure 3H).

### 3.3. High Altitude Increases NOx in Fetal and Maternal Blood

**Figure 4:**
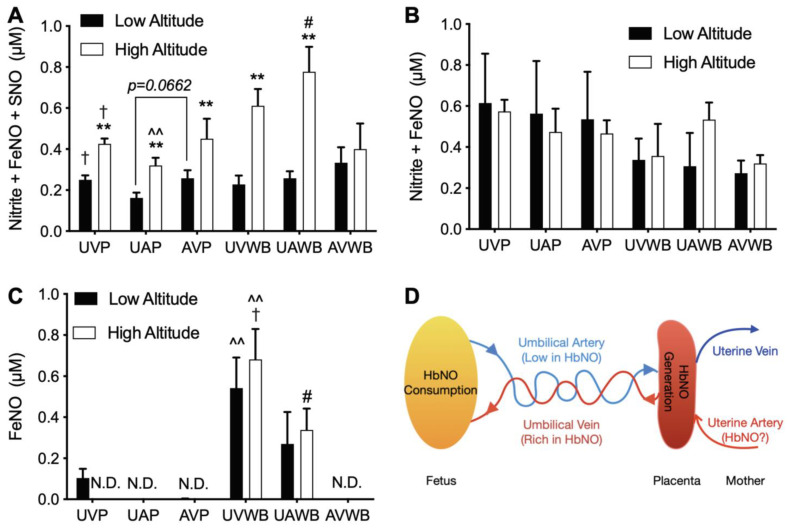
NOx levels in blood from high and low altitude pregnant sheep. NOx were measured by chemiluminescence NO analyzer with various purge vessel reagents. (**A**) I_3_ (measures nitrite, FeNOs, and SNOs). (**B**) FeCN/HAc (nitrite and FeNOs). (**C**) FeCN/PBS (FeNOs). (**D**) Proposed diagram for dynamic homeostasis of HbNO in gestation. UVP: umbilical vein plasma; UAP: umbilical artery plasma; AVP: adult vein plasma; WB: whole blood. N.D.: not detectable. Two-way ANOVA with Tukey’s post-hoc tests for plasma or WB. * = *p* < 0.05 high vs. low altitude; # = *p* < 0.05 fetus (UA) vs. mother (AV); † = *p* < 0.05 umbilical artery vs. vein; ^ = *p* < 0.05 plasma vs. WB. *p* value for paired *t* test in (**A**). One symbol = *p* < 0.05, two symbols = *p* < 0.01.

Four assays that are capable of detecting different NO species were utilized (Figure 4). Therein, the results of the VitC/HAc assay in blood were incomplete and thus are not shown.

In comparison to the low altitude, the I_3_ assay (nitrite, FeNOs, and SNOs) detected increased NOx at high altitude in plasma and whole blood samples from both umbilical artery and vein and also in maternal venous plasma (Figure 4A). As the gold standard assay for bioactive NOx including nitrite, these I_3_ results suggest a significant increase in NO bioavailability in gestation at high altitude, although no such increase was observed by the FeCN/HAc (FeNO and nitrite) or FeCN/PBS (FeNO) assays (Figure 4B–C).

In the I_3_ and FeCN/PBS assays, NOx gradients were demonstrated between fetus and mother (for example, fetal vs. maternal whole blood; Figure 4A,C), umbilical artery and vein (UAP vs. UVP), and plasma and RBCs (UVP vs. UVWB and UAP vs. UAWB), suggesting a complex dynamic homeostasis of NO species among these circulatory compartments in gestation (Figure 4A,C). Notably, in the FeCN/PBS assay, the FeNO levels in high altitude sheep were highest in umbilical venous whole blood, lower in umbilical arterial whole blood, and not detectable in maternal venous whole blood (Figure 4C). These results suggest the generation and efflux of FeNO from the placenta with consumption of FeNO by the fetus (Figure 4D).

### 3.4. High Altitude Increases NOx in Fetal and Maternal Aorta

**Figure 5:**
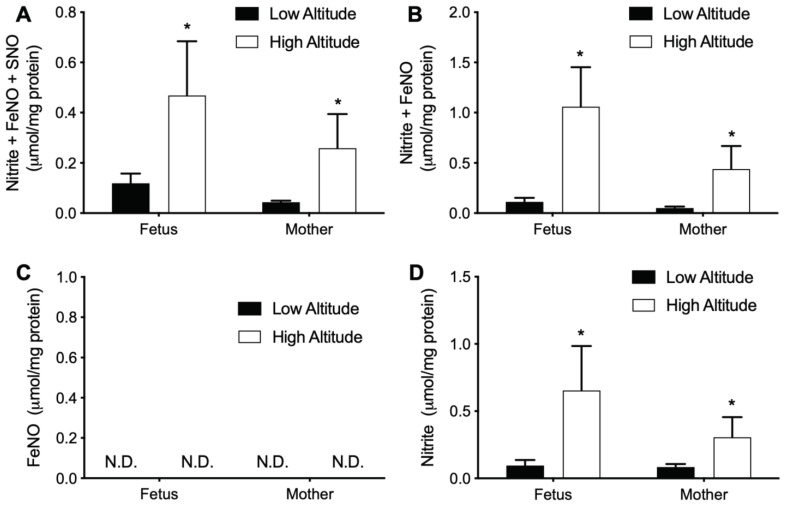
NOx levels in aorta homogenates from high and low altitude pregnant sheep. NOx were measured by chemiluminescence NO analyzer with various purge vessel reagents. (**A**) I_3_ (measures nitrite, FeNOs, and SNOs). (**B**) FeCN/HAc (nitrite and FeNOs). (**C**) FeCN/PBS (FeNOs). (**D**) VitC/HAc (nitrite only). Measurements were normalized to protein concentrations in the homogenates. N.D. = not detectable. Two-way ANOVA. * = *p* < 0.05 high vs. low altitude.

We then measured the effects of high altitude on NOx in the aorta (Figure 5). Measurements with I_3_ (nitrite, FeNOs, and SNOs), FeCN/HAc (nitrite and FeNOs), and VitC/HAc (only nitrite) assays all showed higher NOx levels in high altitude in both fetuses and mothers, while the differences observed between fetuses and mothers were not significant. No FeNO was detected in either fetal or adult, high or low altitude samples with the FeCN/PBS assay.

### 3.5. Effects of High Altitude on Nitrotyrosine and Antioxidant Capacity in Aorta

**Figure 6:**
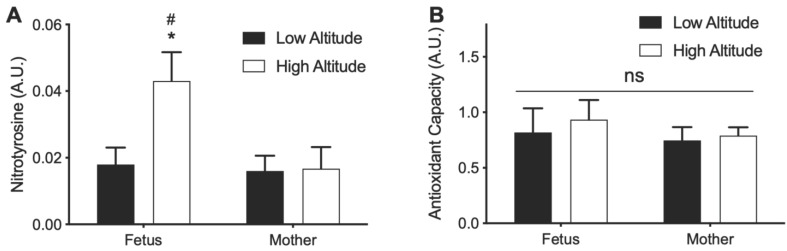
Nitrotyrosine and antioxidant capacity in aorta from high- and low-altitude pregnant sheep. (**A**) Nitrotyrosine. (**B**) Antioxidant capacity. Measurements were normalized to protein concentrations in the homogenates. Two-way ANOVA with Sidak’s post-hoc tests. * = *p* < 0.05 high vs. low altitude. # = *p* < 0.05 fetus vs. mother.

The increase in NOx and decrease in CIP in high altitude aortas led us to measure the levels of nitrotyrosine and antioxidant capacity in aortic homogenates. Aortic nitrotyrosine level in the high-altitude fetus was higher than that in the low-altitude fetus and the high-altitude mother (Figure 6A). However, no significant difference was observed in antioxidant capacity between high- and low-altitude fetus and mother (Figure 6B).

## 4. Discussion

The thin air at high altitude presents a formidable challenge to human physiology. Gestation at high altitude is associated with increased risk of illness [44]. Although many adaptations have been described in both the fetus and mother [1], the effects on iron homeostasis and NO, both of which are under the influence of O_2_ at multiple levels, have not been characterized.

By comparing pregnant ewes from high and low altitudes (3801 and 300 m), the current study demonstrated a systemic redistribution of iron in high altitude fetuses as manifested by depletion of the CIP and a decrease of total iron in the aorta, a non-hematopoietic tissue, and an increase of the NTBI and total iron in the plasma. This study also found significant increases in circulatory and aortic tissue NOx in both fetuses and mothers at high altitude. In addition, the NOx gradients observed between fetus and mother, umbilical artery and vein, and plasma and RBCs, demonstrated a complex dynamic homeostasis of NOx among these circulatory compartments. Specifically, the results are consistent with the recent work that suggests the placenta serves as a source of FeNOs to the fetus [27], and that a measurable portion of these FeNOs in the RBCs are consumed within one circulatory time from the umbilical vein to the umbilical artery.

It has been widely held that the fetus behaves as a parasite for maternal iron in that the fetus is able to acquire iron from the mother irrespective of her iron status [45]. In contrast to this notion, the current experiments demonstrated that high altitude only decreased the total iron in fetal aorta but did not alter the total iron in maternal aorta, suggesting that high altitude results in iron deficiency in the fetus but not in the mother. Nevertheless, in striking contrast to the aorta, both the NTBI and total iron levels in fetal plasma were increased by high altitude, suggesting that high altitude results in iron overload in the fetus. This simultaneous deficiency of iron in aorta and overload of iron in plasma observed in high-altitude fetuses demonstrated an intriguing systemic redistribution of fetal iron. Reports on systemic iron redistribution are quite rare. While several diseases such as chronic anemia, hypotransferrinemia, and aceruloplasminemia have been related to systemic iron redistribution, deprivation rather than accumulation of plasma iron was found under these conditions [39,46,47,48]. It has been reported that high altitude hypoxia caused a net loss of skeletal muscle iron associated with a high rate of iron uptake by bone marrow for erythropoiesis [49]. Given the 21% increase in hemoglobin concentration in fetuses exposed to high altitude [7], it is reasonable to speculate that the observed iron redistribution in the present study was also an erythropoietic response to hypoxia. Because the aorta is a non-hematopoietic tissue, these results suggest the mobilization of iron from non-hematopoietic tissues into the circulation to be utilized for erythropoiesis in the fetus to cope with the high-altitude hypoxia. Notably, hepcidin has been proposed to be a central regulator of systemic iron balance, with maternal plasma hepcidin regulating placental iron transport while the fetal plasma hepcidin operates autonomously to regulate fetal iron homeostasis, including erythropoiesis [50,51]. It is generally accepted that hypoxia decreases hepcidin [52]. Consistent with this notion, maternal hepcidin was decreased at high altitude, favoring iron transport across the placenta to the fetus. However, the fetal hepcidin was unaltered by high altitude, which would suggest that the systemic redistribution of iron observed in high altitude fetuses was not mediated by hepcidin.

The iron deficiency observed in the aorta of high-altitude fetuses is in contrast to the well-documented increase of cellular iron availability in response to hypoxia [18,52]. Cellular iron availability is regulated by the balance of iron uptake and export. Hypoxia has been shown to upregulate circulating levels of transferrin and ceruloplasmin, which increases the uptake of iron into cells [52]. In contrast, the current study found decreased blood transferrin and decreased RBC membrane-bound ceruloplasmin in high altitude fetuses and mothers. It is possible that these alterations in circulating transferrin and ceruloplasmin contribute to decreased iron uptake to the aorta in the high-altitude fetuses. Another possibility is that fetal aorta cells may have enhanced rates of iron export at high altitude. Indeed, hypoxia is known to increase ferroportin, the only known cellular iron exporter, by suppressing hepcidin [16,53]. However, contrary to this possibility, neither aortic ferroportin nor plasma hepcidin were altered in fetuses at high altitude. Alternatively, it is important to note the possibility that cellular iron, particularly the CIP, may also be actively exported as DNICs, which are formed via incorporation of iron and NO [14,18,54]. The depletion of the aorta CIP and increased aorta NO availability in high-altitude fetuses are both supportive of this possibility. Although the current assays failed to detect DNICs in aorta homogenates from either high- or low-altitude fetuses (Figure 5C), it is also worth noting that these null results should be taken with caution due to the likelihood of DNICs degrading to other forms of NOx [28,55] during freeze-thaw cycles for storage or the ~2 h tissue homogenization process. The mechanisms driving the iron deficiency in the aorta of high-altitude fetuses merits further investigation.

Many signals measured by EPR are of value as indices of redox status. For instance, metHb and ferric heme proteins are products of heme oxidation, ceruloplasmin is a superoxide scavenger and also an antioxidant enzyme, and free radicals serve as a direct measurement of reactive species. As described in the Results section, all these measurements in blood were altered in the direction of increased oxidative stress at high altitude, particularly in the fetus. In addition, the NTBI, a circulatory marker of oxidative stress, was upregulated at high altitude in both the fetus and mother. One might argue that the increase in MetHb and NTBI was simply due to the hypoxia-induced increase in hemoglobin concentration. However, although total hemoglobin concentrations were not measured in the current study, previous work with this animal model would predict a ~21% increase in the hypoxic fetuses [7], which is significantly less than the >2-fold increase observed for MetHb and the NTBI in the current animals. Furthermore, plasma nitrotyrosine, another marker of oxidative stress, was also increased in the fetuses at high altitude, although no significant alteration was observed in the mother. Together, these circulatory measurements suggest that gestation at high altitude is associated with increased oxidative stress, especially in the fetus.

In contrast to the circulatory measurements suggesting increased oxidative stress at high altitude, the depletion of the CIP in the aorta of both fetus and mother at high altitude would go against the production of reactive species [56]. Likewise, antioxidant capacity in the aortic homogenates was not affected by high altitude in either the fetus or the mother, again suggesting that high altitude does not result in oxidative stress in the aorta. These results are consistent with previous metabolomic findings in this animal model wherein high altitude did not alter markers of oxidative stress or antioxidant thiol-metabolism pathways in fetal carotid arteries [57]. Therefore, the effects of high-altitude hypoxia on fetal redox status appear to be different in the blood than in the aorta.

The current study found that high altitude resulted in significant increases in NOx levels both in circulation and in the aorta of both fetuses and mothers. These results are consistent with the majority of previous reports, which propose the increase of NO species as a compensating response to high altitude-induced hypoxia stress [1,24,58,59]. In addition, contrary to a report that NOx species decrease in pregnant women at high altitude [26], the current results suggest that the increase of NOx at high altitude occurs irrespective of pregnancy and happens to both fetus and mother. Notably, unlike previous reports, the I_3_ assay detected the bioactive nitrite, FeNO, and SNOs but not nitrate, which is not reflective of endothelial function and is also orders of magnitude more abundant than the bioactive NO metabolites [14]. In addition, it is also important to note that the current measurements were performed with fresh blood samples and freshly homogenized aorta samples, in contrast to previous reports. Many of the commonly assayed bioactive NO species are under dynamic equilibria during sample handling which can affect their final measured concentrations [15,16,17]. Therefore, the current measurements with fresh samples were more likely to reflect actual in vivo NOx concentrations. As a result of these advantages, the observation of increased NOx levels at high altitude in the current study provides stronger evidence than previous reports that NO bioavailability is increased in response to the hypoxic stress of high altitude. The identity of the specific NOx species that make up the increases in response to hypoxia remains unclear. Based on the selectivity of the various chemiluminescence reagents, it appears that much of the increase can be attributed to SNOs. However, caution is warranted for this assumption. Notably, although the chemiluminescence NOx assays represent state-of-the-art methodology [28], a mass balance of the measured species could not be achieved by comparison of the various selective reagents. For example, the levels of nitrite + FeNO measured by the FeCN/HAc assay were often double the levels of nitrite + FeNO + SNOs measured by the I_3_ assay.

The increase of NOx at high altitude has been proposed to result from the upregulation of eNOS via the hypoxia-induced factor 1alpha (HIF-1α) pathway [24,25]. However, previous examination in the high-altitude model found eNOS levels to be unaltered, at least in the pulmonary vasculature [60]. Furthermore, while HIF-1α levels were increased during the initial period of exposure to hypoxia, levels returned to baseline after chronic exposure [1]. Decreased eNOS levels have also been reported in fetal sheep brains and guinea pig hearts after exposure to hypoxia [61,62], raising further questions about the role of eNOS in the increase of NOx at high altitude. The homeostasis of NOx is a complicated balance of NO production by NOS, dietary intake, and oral microbiome activity, various pathways of metabolism, many of which are O_2_-sensitive, and renal excretion [20]. Further study is needed to investigate the mechanisms underlying the increase of NOx at high altitude.

An intriguing finding of the current study is the NOx gradients observed between fetus and mother, umbilical artery and vein, and plasma and RBCs. These NOx gradients are of significant physiological importance in that they demonstrate complex dynamic homeostasis of different NOx species across these circulatory compartments. For instance, it is worth noting the FeNO gradients between umbilical venous whole blood (highest), umbilical arterial whole blood (lower), and maternal venous whole blood (null), which suggested the placental generation and efflux into the fetal circulation. The arteriovenous gradient of FeNO species also suggests there is dynamic production of this specific NOx species in the placenta as well as consumption by the fetus at rates that result in significant turnover of the overall FeNO pool within the circulatory transit time through the body. Gradients of various NOx species between fetus and mother, artery and vein, and plasma and blood/RBCs have been reported previously [63,64,65,66,67,68]. However, no consensus has been reached, in large part due to methodological concerns about the specificity of the assays for various NOx species, leading to debate over which NOx species, if any, is the prevalent bioactive metabolite of NO in the circulation [69,70]. The current results not only confirm the previous report that placenta excretes FeNO into plasma [27], but also further demonstrated that most of the placental efflux of FeNO is within the RBCs. It is worth noting that the fetal arterio-venous difference of placental-derived FeNO would suggest that FeNO levels should fall rapidly at birth when the umbilical cord is ligated. This may play a significant role in the rapid increase in systemic vascular tone that occurs in the newborn within minutes after birth [27]. In addition, our recent work demonstrated that the placenta efficiently converts nitrite into heme-iron nitrosyl complexes [66], an endogenous placental NOx that may be altered in pregnancies complicated by preeclampsia [28]. Therefore, the fetal RBC FeNO effluxed from placenta at high altitude probably was HbNO (Figure 4D).

This study has several limitations. First, non-pregnant ewes were not studied. The comparisons between pregnant and non-pregnant animals at high and low altitudes would provide more information about the impact of high-altitude hypoxia on gestation. Second, NOx measurements of blood samples from uterine artery and vein are missing. These measurements, together with those of blood from the umbilical artery and vein in the absence and presence of NOS inhibition may help identify the source of HbNO released by the placenta. Third, further study is needed to verify the null results of DNICs in the aorta, with special attention paid to the effects of sample processing, including the effects of freeze-thaw and tissue homogenization, on the stability of DNICs. Finally, further validation and development of the chemiluminescence NOx assays is warranted in the future.

## 5. Conclusions

Overall, this study suggests exposure of the fetus to chronic high-altitude hypoxia during gestation results in the utilization of non-hematopoietic tissue iron for erythropoiesis and increased NO bioavailability, which may offset the hypoxic stress of high altitude. Findings of this research may help development of treatments to mitigate the adverse health effects of high altitude, particularly in pregnant women and fetuses, and pregnancies complicated by conditions such as pre-eclampsia and intrauterine growth restriction.

## Data Availability

Data is contained within the article.
